# Evidence of molting and the function of “rock-nosing” behavior in bowhead whales in the eastern Canadian Arctic

**DOI:** 10.1371/journal.pone.0186156

**Published:** 2017-11-22

**Authors:** Sarah M. E. Fortune, William R. Koski, Jeff W. Higdon, Andrew W. Trites, Mark F. Baumgartner, Steven H. Ferguson

**Affiliations:** 1 Department of Zoology and Marine Mammal Research Unit, Institute for the Oceans and Fisheries, University of British Columbia, Vancouver, British Columbia, Canada; 2 Biology Department, Woods Hole Oceanographic Institution, Woods Hole, Massachusetts, United States of America; 3 LGL Limited, King City, Ontario, Canada; 4 Higdon Wildlife Consulting, Winnipeg, Manitoba, Canada; 5 Fisheries and Oceans Canada, Freshwater Institute, Winnipeg, Manitoba, Canada; Institute of Deep-sea Science and Engineering, Chinese Academy of Sciences, CHINA

## Abstract

Bowhead whales (*Balaena mysticetus*) have a nearly circumpolar distribution, and occasionally occupy warmer shallow coastal areas during summertime that may facilitate molting. However, relatively little is known about the occurrence of molting and associated behaviors in bowhead whales. We opportunistically observed whales in Cumberland Sound, Nunavut, Canada with skin irregularities consistent with molting during August 2014, and collected a skin sample from a biopsied whale that revealed loose epidermis and sloughing. During August 2016, we flew a small unmanned aerial system (sUAS) over whales to take video and still images to: 1) determine unique individuals; 2) estimate the proportion of the body of unique individuals that exhibited sloughing skin; 3) determine the presence or absence of superficial lines representative of rock-rubbing behavior; and 4) measure body lengths to infer age-class. The still images revealed that all individuals (n = 81 whales) were sloughing skin, and that nearly 40% of them had mottled skin over more than two-thirds of their bodies. The video images captured bowhead whales rubbing on large rocks in shallow, coastal areas—likely to facilitate molting. Molting and rock rubbing appears to be pervasive during late summer for whales in the eastern Canadian Arctic.

## Introduction

The skin (epidermis) and hair (keratinized epidermal cells) of marine mammals are specially adapted for life in an aquatic environment. The periodic shedding of part or all of their outer layer of epidermal covering, which is then replaced by new growth [1] has been well studied for seals and sea lions—which molt annually to repair and renew their skin and pelt [2–8]. In contrast, whales, dolphins and porpoises are generally thought to continuously shed and replace their epidermis [9,10]. However, this may not be the case for Arctic species that experience pronounced changes in environmental conditions by seasonally occupying uncharacteristically warmer areas such as estuaries and fiords [11].

Beluga whales (*Delphinapterus leucas*) and most likely narwhal (*Monodon monoceros*) (e.g., Inuit hunter observation [12]) undergo a seasonal epidermal molt during summer. The beluga whale molt appears to be facilitated in part by the warm and low salinity environmental conditions found in seasonally occupied estuaries [11,13]. The elevated water temperatures are postulated to influence the growth and turnover of epidermis by increasing metabolic activities [11] or provide an evolved physiological cue (e.g., daylight [14]). Furthermore, physical features of estuaries such as gravel bottoms provide an abrasive surface to rub against and expedite exfoliation [15].

In contrast to what is known about molting for beluga whales, little is known about this phenomenon in bowhead whales (*Balaena mysticetus*). It is known, for example, that the structure of epidermal, dermal and hypodermal layers of balaenid whales (bowhead and right whale, *Eubalaena* sp.), closely resembles that of odontocete species that are known to slough skin (e.g., beluga whales) and differs from the more closely related balaenopterids (e.g., fin, blue, and sei whale) [1]. Furthermore, southern right whale (*Eubalaena australis*) calves are known to shed multiple layers of their epidermis [16], which is similar to beluga calves that conduct a multilayered molt to remove fetal epidermis [17]. Histological analysis has revealed that bowhead whales belonging to the Okhotsk Sea population molt during summer months while occupying a warm, shallow bay in the Shantar Achipelago [18]. However, it is not known whether other populations of bowhead whales, such as the Eastern Canada-West Greenland (EC-WG) population, undergo a seasonal molt or whether they molt continuously and whether the molting process is similar for all age classes of whales.

## Materials and methods

**Fig 1:**
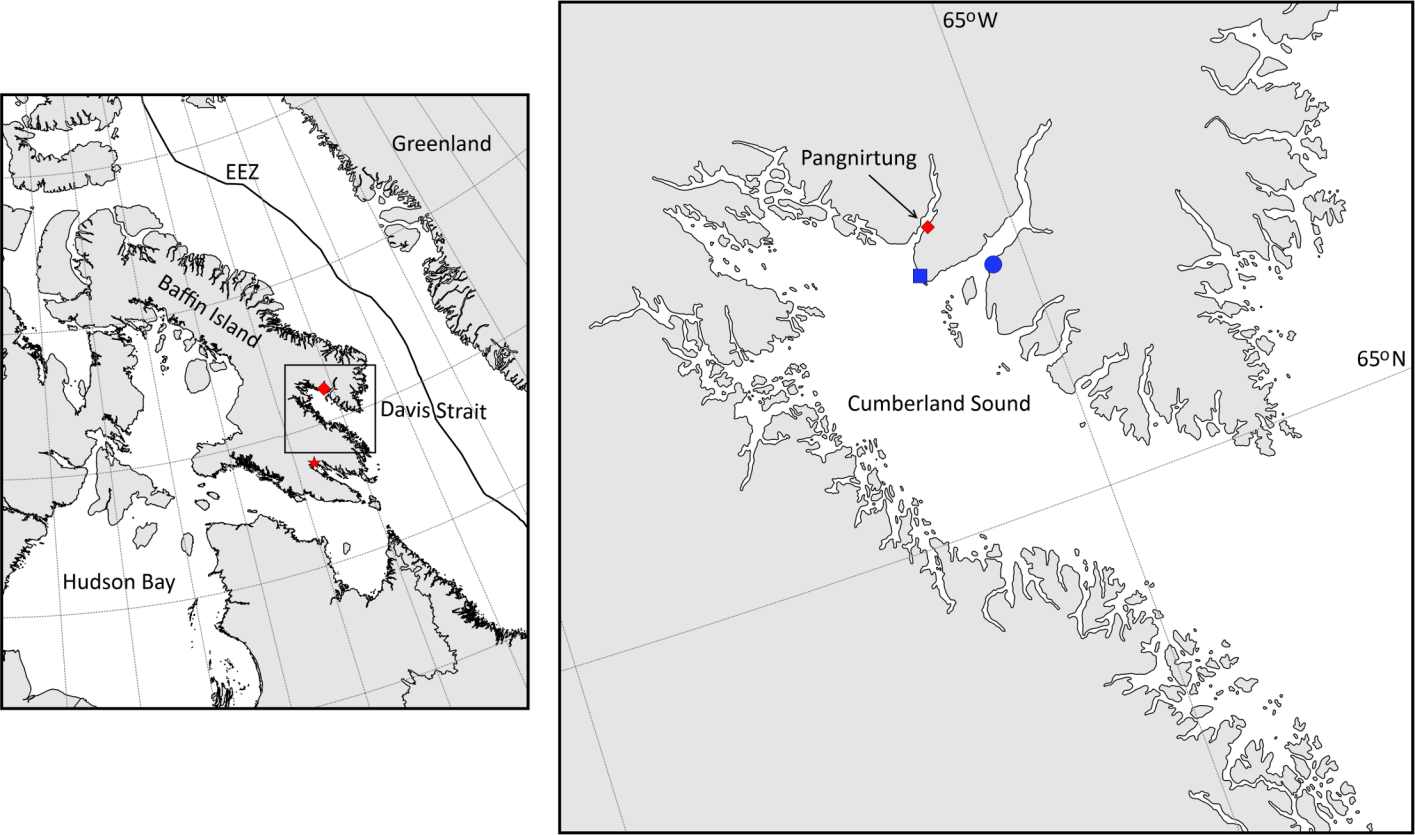
Locations of fieldwork conducted in the eastern Canadian Arctic showing Baffin Island, Canada (left panel), and Iqaluit, the capitol of Nunavut (designated with a black ↆ) and Pangnirtung, a community located in Cumberland sound (red ◆). The inset map of Cumberland Sound shows where bowhead whale (*Balaena mysticetus*) observations were made in a small bay in Kingnait Fiord (blue ●) in 2014 from a vessel and from sUAS in Brown Harbour (blue ■), Pangnirtung Fiord and Kingnait Fiord in 2016. The polygon shapefiles and polyline shapefile used to generate this map were accessed through Esri Canada and were licensed through Natural Resources Canada for free distribution.

We opportunistically made boat-based sightings of EC-WG bowhead whales in Cumberland Sound, Nunavut, Canada—specifically in Kingnait Fiord (located on the northeast side of Cumberland Sound Fig 1)—on five days from 13–21 August, 2014. As a result of these preliminary observations, a directed study to test the hypothesis that bowhead whales use Cumberland Sound in part for molting during summer months was carried out in August 2016.

Zooplankton samples were opportunistically collected during August 2014 from surface waters (0.5 m) near bowhead whales, and also following an unusual observation of bowhead whales in shallow, coastal waters in Kingnait Fiord. All samples were collected using 333-micrometer (μm) conical mesh net (30 cm in diameter) fitted with a General Oceanics helical flow meter. The zooplankton net was sprayed down with a hose using seawater to collect the sampled organisms in an attached cod-end bucket once it was brought onboard the boat. Once organisms were no longer visible in the zooplankton net, the cod-end bucket samples were filtered through a 333 μm mesh sieve and transferred to a 250 mL sample jar and preserved in 5% buffered formalin solution for identification.

Boat-based and aerial sightings of bowhead whales were made in Pangnirtung Fiord, Brown Harbour (located between Pangnirtung Fiord and Kingnait Fiord, Fig 1) and Kingnait Fiord from 7–31 August 2016. High-resolution aerial images (n = 1143) and video were obtained of encountered whales using a small unmanned aerial system (sUAS), the DJI Phantom 3 Professional. The sUAS was equipped with a global positioning system (GPS) and altimeter that allowed for the whale’s position and the sUAS altitude to be automatically recorded when each image was captured. The sUAS was flown at an average altitude of 12.9 m (±5.4 SD) with a maximum distance of 1000 m from the survey vessel, and was hand-deployed and hand-retrieved from the ~8 m aluminum vessel. Flight times lasted ~8–12 min. The sUAS data were collected under Special Flight Operation Certificate File Number 5812-11-682, ATS 16-17-00014027, RDIMS 12044419 and approved by the University of British Columbia Animal Care Committee (Animal Care Amendment A14-0064-A002). Bowhead whale behavioral data were collected under permit Department of Fisheries and Oceans License to Fish for Scientific Purposes S-16/17 1005-NU and Animal Use Protocol FWI-ACC-2016-09.

**Fig 2:**
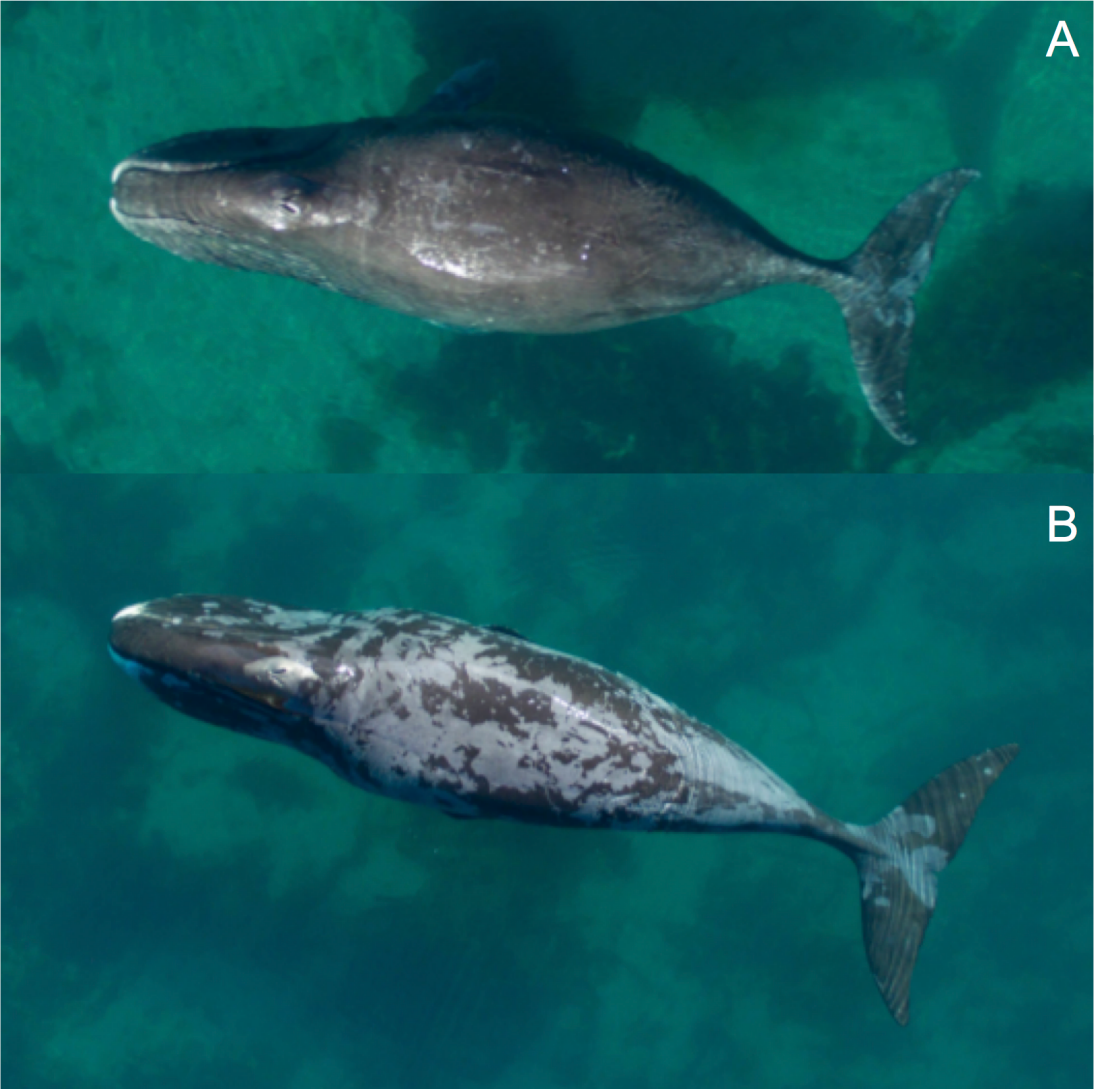
Example of an animal with nearly no sloughing skin (i.e., proportion of body with sloughing skin = <33%) (**A**) and another bowhead whale with a high degree of sloughing (>66% of body) and a blotchy skin type (**B**).

**Fig 3:**
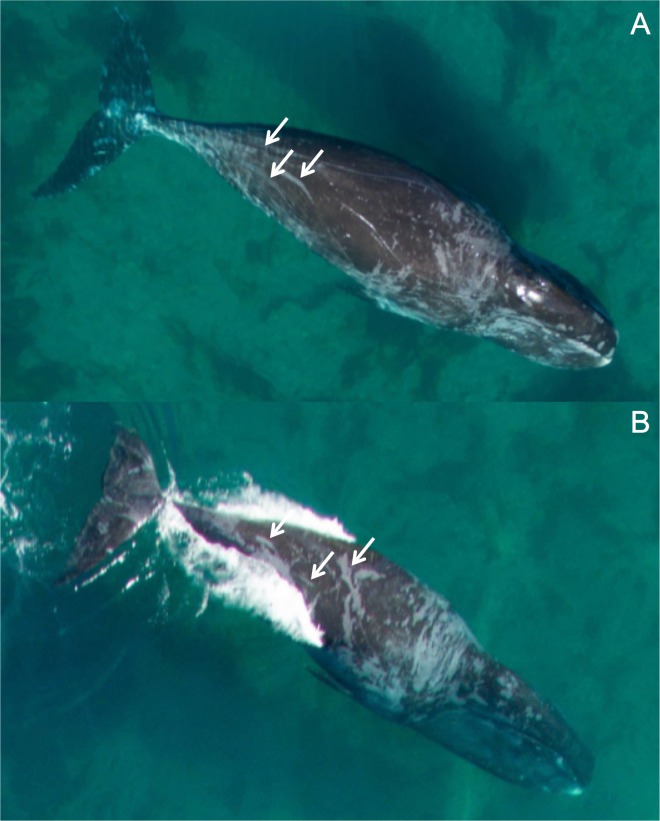
Example of (**A**) a bowhead whale with thin and sharp light gray lines and (**B**) of a whale with shorter and wider gray lines, that both likely reflect prior rock-rubbing behavior.

Still sUAS images of bowhead whales were used to determine unique animals during August 2016 as well as their body lengths and skin conditions. Individual animals were identified using well-established permanent black and white dorsal patterns [19]. The markings used to identify unique individuals included: 1) white scars on their body attributed to breaking sea ice, encounters with fishing gear or from interactions with killer whales (*Orcinus orca*) (i.e., killer whale rake marks [20]; and 2) white pigmentation found on the dorsal flukes, caudal peduncle and lower jaw. Body length measurements (distance between snout and fluke notch) were made for animals that were photographed with the vessel (an object of known size used for calibration) in the same frame or another animal of known size (i.e., previously photographed with the vessel). The measuring tool in Adobe Photoshop CS6 extended was used to measure body length. Skin condition was also assessed from still images to determine: 1) proportion of the body that contained sloughing skin (0 = none, 1 = <33%, 2 = 33–66%, 3 = >66% and <100% and 4 = 100%; Fig 2); and 2) the type of sloughing (0 = none, 1 = light gray lines across the body likely caused by rock rubbing, 2 = irregular patches of gray sloughed skin, 3 = smooth gray body attributed to complete or near complete sloughing; Fig 3). The presence of gray tissue is indicative of new skin growth—based on our observations and those of bowheads molting in the Okhotsk Sea [18]. Three people independently scored each animal, and agreement between at least two people was required to obtain a final score.

We opportunistically collected oceanographic data during August 2016 to evaluate the physical properties of Kingnait Fiord. Vertical profiles of the water column were made using a seabird SBE19Plus conductivity, temperature, and depth (CTD) profiler. CTD data were corrected using Seabird software and temperature and salinity plots were generated using the downcast data for each cast (n = 86).

## Results and discussion

### 2014 Observations

**Fig 4:**
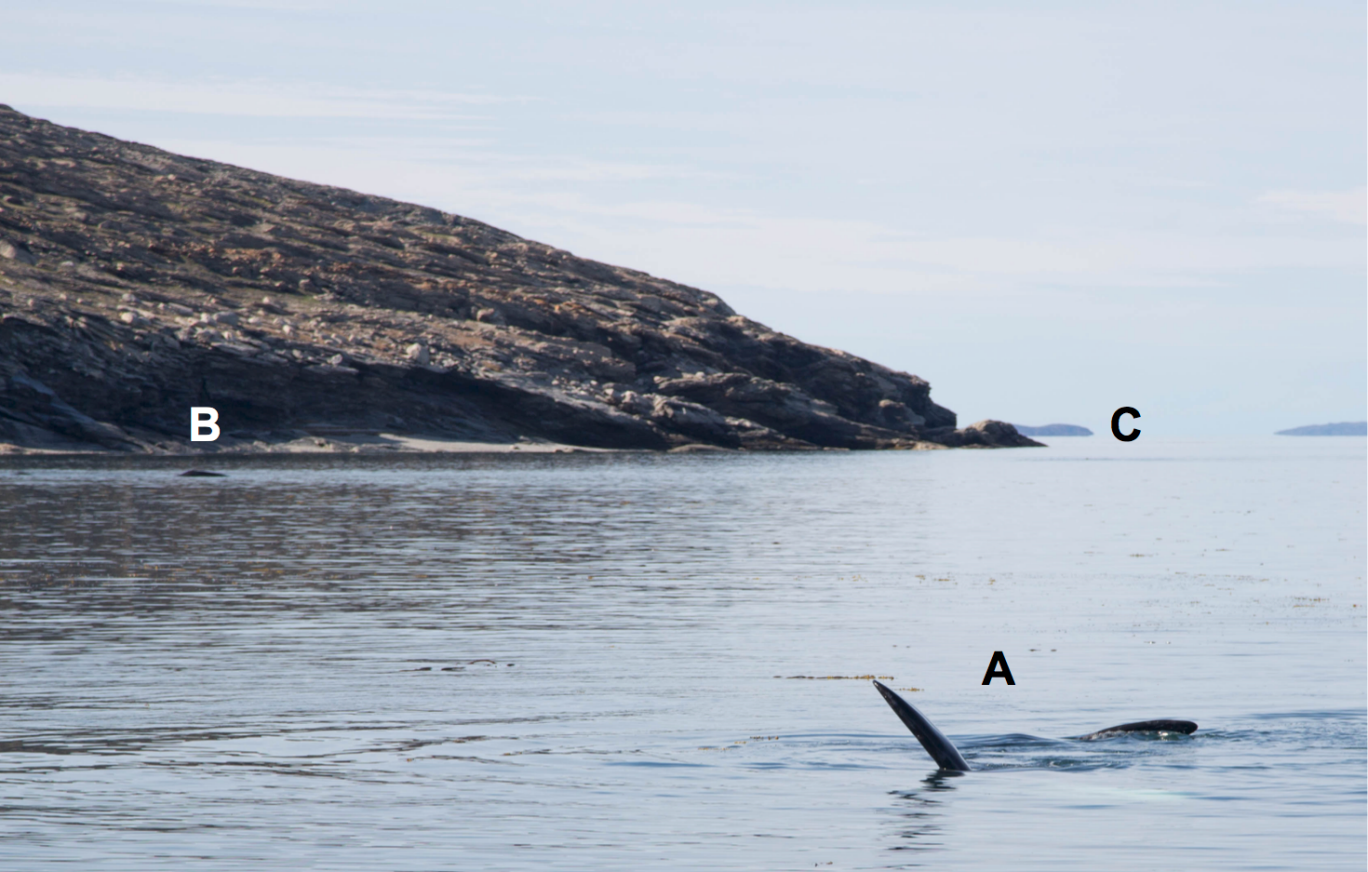
Example of observed behavior and relative distribution of three individual bowhead whales (**A-C**) inside the bay in Kingnait Fiord (2014). Whale (**A**) is resting close to shore with its head out of the water, (**B**) is on its back with pectoral flippers extended, and (**C**) is breaking the surface of the water in the distance.

**Fig 5:**
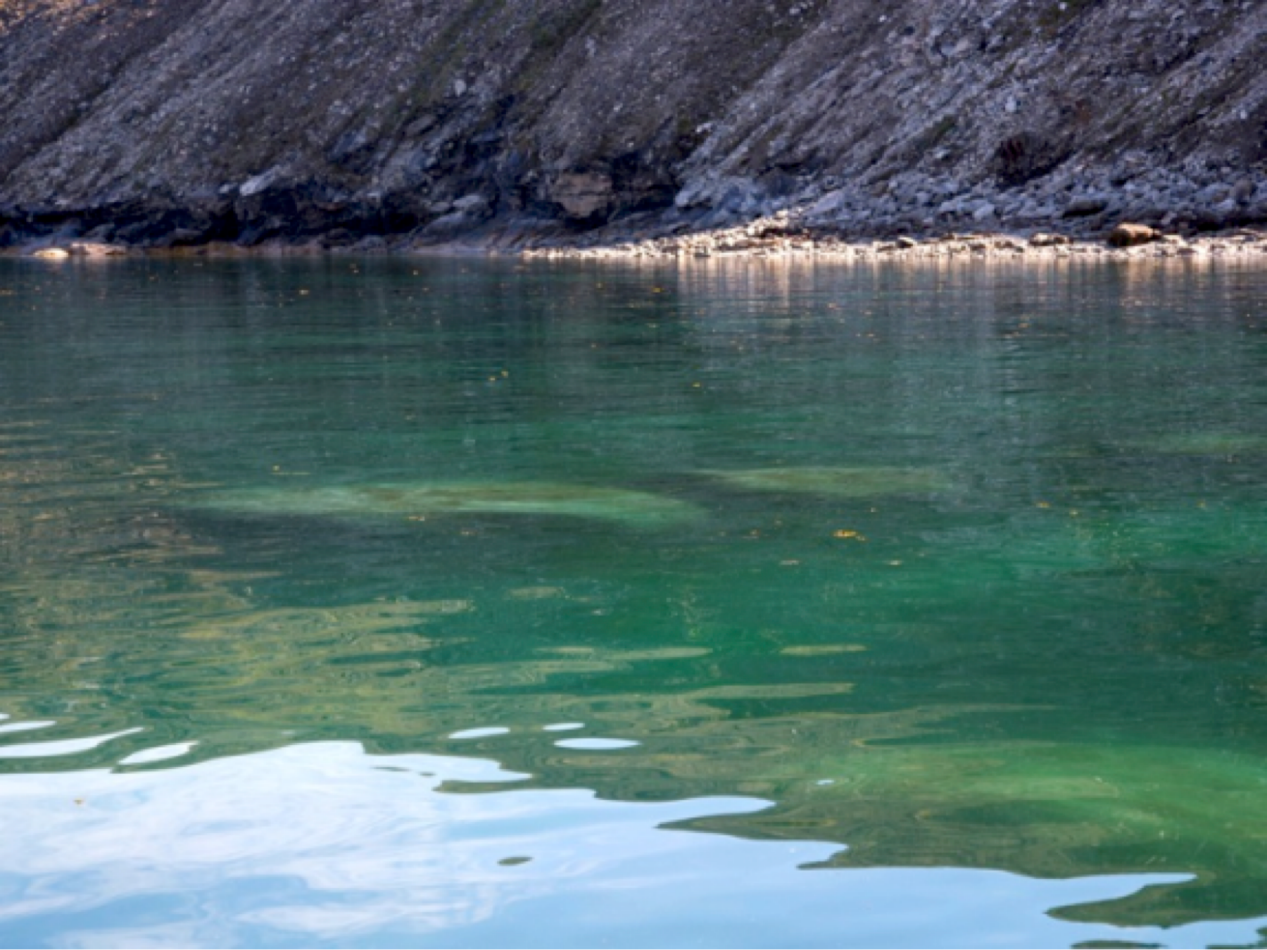
Large boulders located in the shallow bay where the bowhead whales (*Balaena mysticetus*) aggregated in Kingnait Fiord, NU, during 2014 and where prey samples were subsequently collected.

Unusual behaviors of bowhead whales in shallow coastal-waters were first noted on 21 August 2014 in Kingnait Fiord when 8–10 bowhead whales were observed frequently rolling onto their sides and backs and lifting their pectoral flippers out of the water (Fig 4). The small, shallow bay was marked with large boulders (65° 55’15.2”N and 65° 17’50.8” W, Figs 4 & 5) and the whales were in ~8 m of water. The animals did not appear to be associated with one another as they were widely distributed throughout the bay and exhibited individually specific behaviors. Several audible vocalizations were heard without a hydrophone from the vessel over the course of the sighting. Net sampling revealed very low zooplankton biomass, particularly for species that are known bowhead whale prey (e.g., calanoid copepods, euphausiids, amphipods), indicating that the whales were not feeding in the bay. There was little likelihood that the whales were feeding because subsequent zooplankton sampling failed to capture bowhead whale prey.

**Fig 6:**
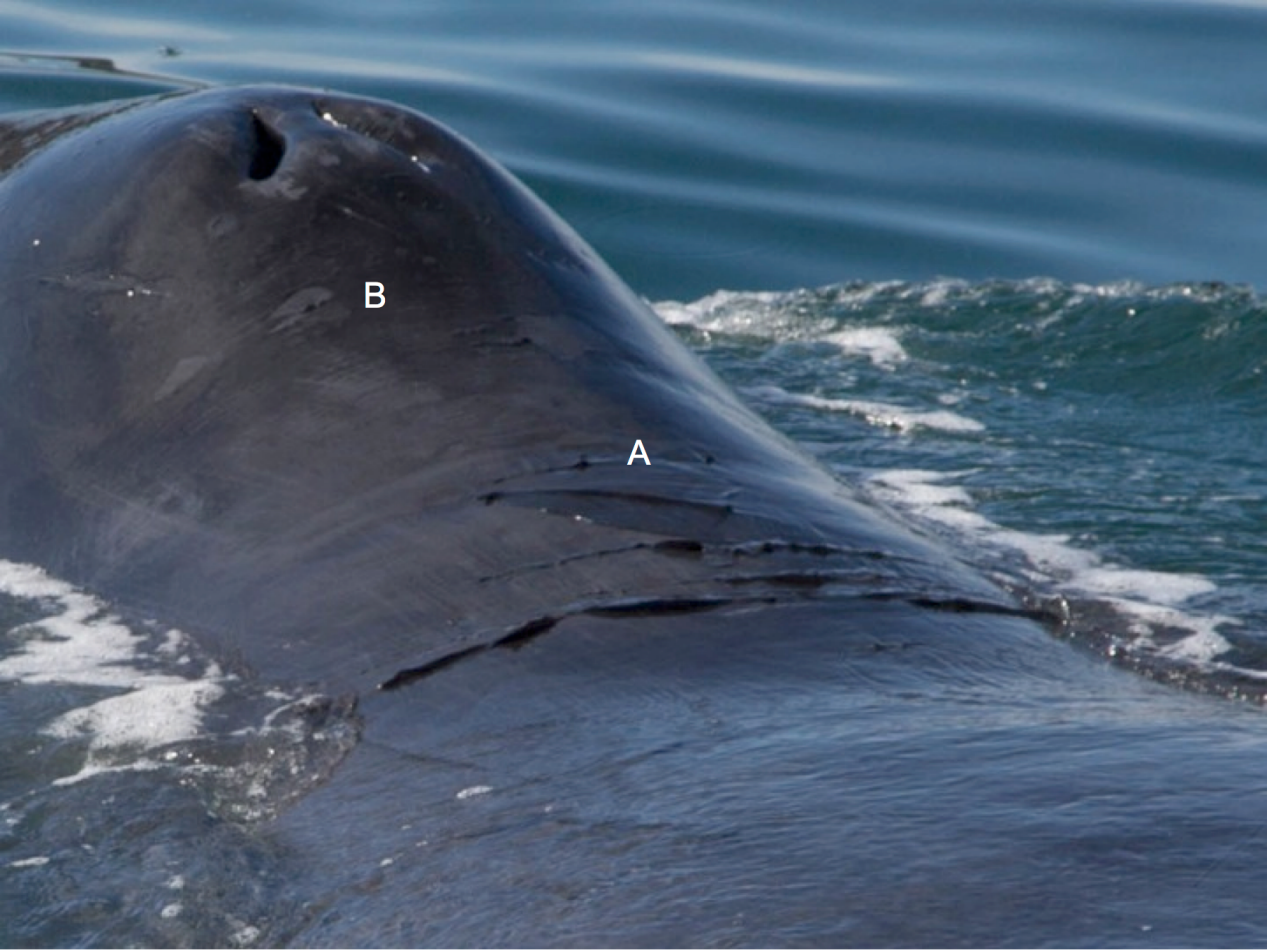
Example of sloughing epidermis (**A**) located behind the blowholes of a bowhead whale (*Balaena mysticetus*) and mottled skin (**B**) found near the blowholes.

Irregularities in bowhead whale skin condition were observed from animals visiting the bay. Photographs taken of two whales before they reached the bay revealed large pieces of loose epidermis. The sloughing epidermis was predominately located posterior to the whale’s blowholes (Fig 6). Animals also presented mottled skin consisting of light gray irregular patches on their heads near the blowholes and on their backs (Fig 6). Furthermore, histological analysis of a sample of loose epidermis from one whale obtained using a crossbow and biopsy dart was consistent with molting. These documented skin irregularities were similar to the histology of biopsy and opportunistic skin collected from bowhead whales in the Okhotsk Sea [18], and provide support that molting occurs during summer based on the timing of collection and histological properties of our bowhead samples [21].

### 2016 Observations

#### Rock rubbing

**Fig 7:**
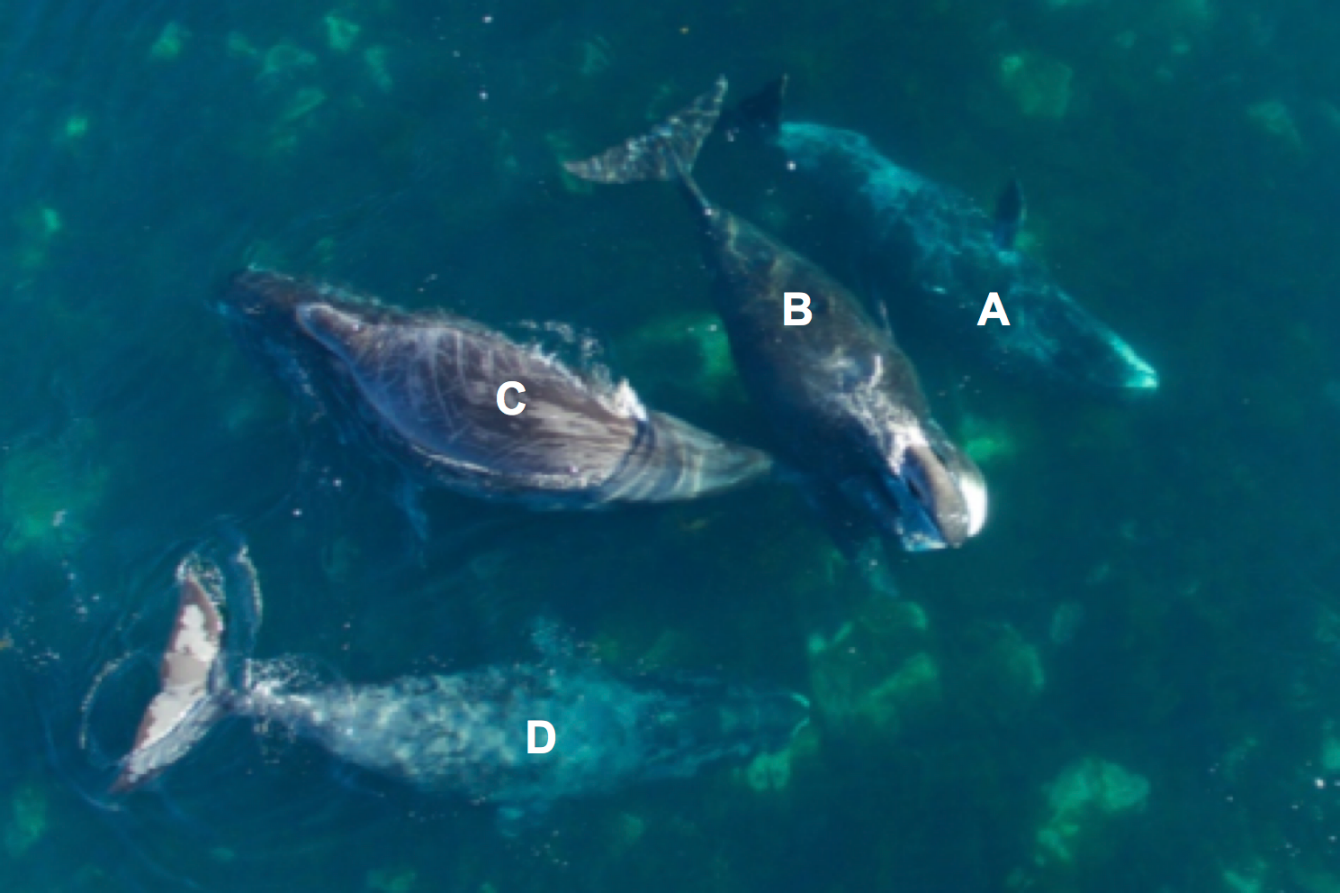
Example of four bowhead whales (*Balaena mysticetus*) with mottled skin rubbing their bodies against boulders in Brown Harbour on 7 August 2016. Animal (**A)** pictured rubbing the right side of its head on a boulder (S1 Movie) and animal (**D**) is using the rocks to exfoliate its chin. Evidence of prior rock-rubbing is apparent for animal (**C**) with long, thin lines running length and width-wise across the body.

Following our initial observations in 2014, we used the sUAS to document four bowhead whales rubbing on large boulders in shallow coastal waters on 7 August 2016 in Brown Harbour (65° 58’31.0” N and 65° 57’19.0” W, Fig 1). While simultaneously filming this rubbing behavior, we made boat-based observations of whales rolling onto their sides with pectoral flippers extended out of the water similar to our prior observations (during summer 2014). The aerial video and high-resolution still images of the animals displaying surface behaviors confirmed that they were rubbing their bodies against rocks (Fig 7 & S1 Movie). The whales were seen rubbing their chins, head, back and sides on a cluster of boulders and had mottled skin with what appeared to be long superficial scratches that ran lengthwise and widthwise along their bodies (e.g., Fig 7 animal C). This rubbing behavior was consistent with previous observations and supported our hypothesis that bowhead whales engage in exfoliation activities during the summer in Cumberland Sound. We presume that rubbing activities caused the linear markings. One animal was observed rock rubbing for a minimum of 8 minutes based on aerial imagery (S1 Movie).

Previous observations of bowhead rock-rubbing behavior have been documented during late summer and early fall in Isabella Bay (Baffin Bay), whereby bowhead whales engaged in “grooming” activities by rubbing on the bottom [22,23]. Similarly, whaling records [24] provide historical support for bowhead rubbing behavior dating back to ~1845, whereby whales found in the bays and inlets of Davis Strait (such as Cape Searl) were referred to as “rock-nose” whales because they would place their head or “nose” close to the shore on a rock [25]. More recently, similar “rock-nose” behavior was observed during an aerial survey of Isabella Bay on 13 September 1979 [26].

Recent Inuit observations of bowhead whales with molting skin during summer were made near Clyde River (nearest community to Isabella Bay) [27]. Whales were also observed circling around a large rock off the coast of Clyde River [27], and were hypothesized to use the rocks for resting purposes [24]. However, the association between EC-WG bowhead whales with molting skin and their physical environment suggests otherwise. The whaling data provide further evidence that they have engaged in rock-rubbing behavior off the coast of Baffin Island for at least hundreds of years.

#### Aerial image analysis

**Fig 8:**
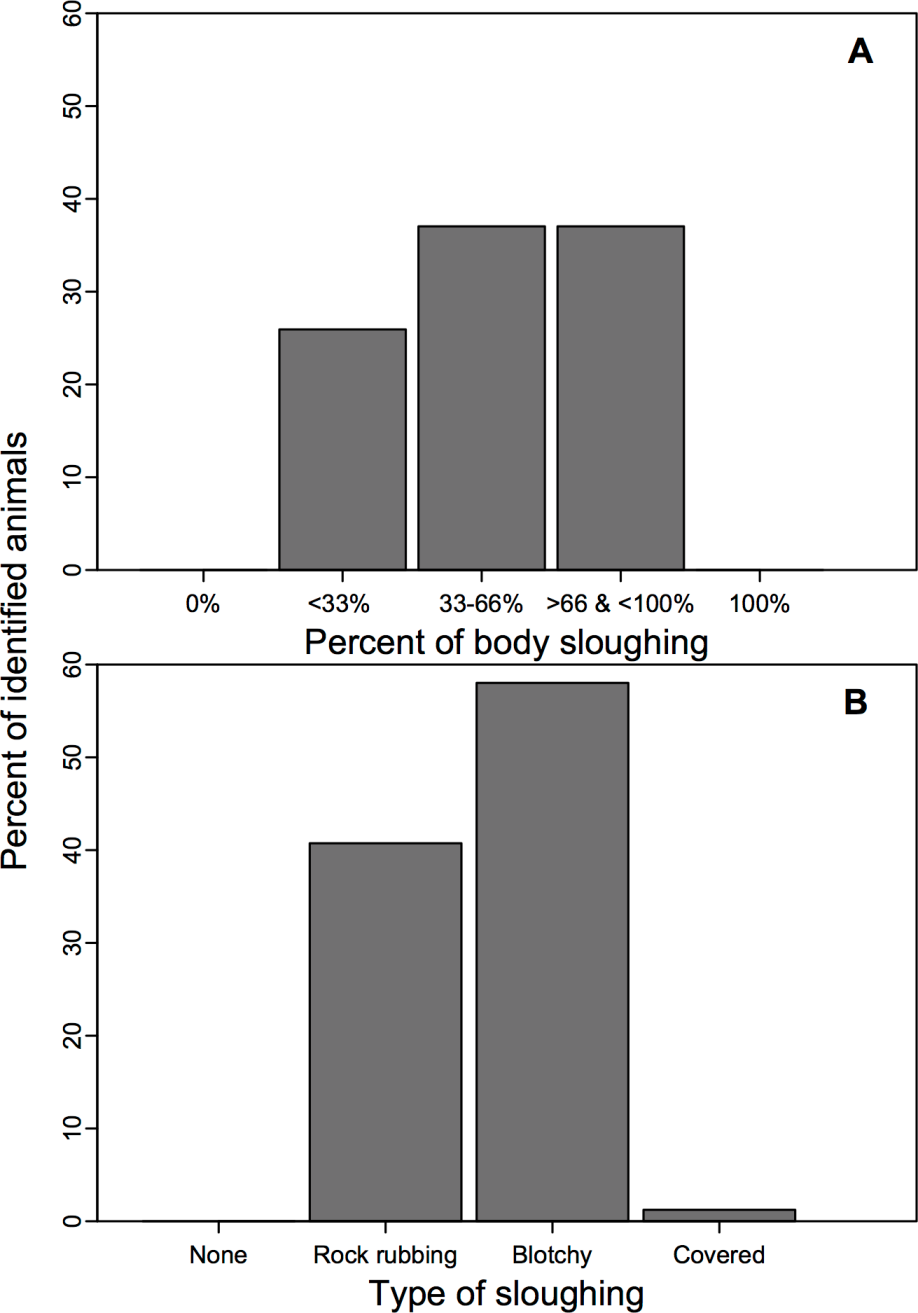
Percentage of photographically identified bowhead whales with sufficient image quality to quantify the amount of the body containing sloughing skin (n = 81) **(A)**. Overall, all animals showed signs of molting in late August, and no animals had bodies with 100% new skin cover. Also shown is type of sloughing skin **(B)** whereby ‘none’ represents animals with no skin irregularities, ‘rock rubbing’ is indicated by animals with both sharp, thin light gray lines and/or wider, less pronounced light gray lines which are likely a result of prior rock rubbing, ‘blotchy’ comprises animals with irregular patches of new epidermis and ‘covered’ includes animals with extensive new skin growth.

Our analysis of high-resolution images from the sUAS indicates pervasive molting for individuals occupying Cumberland Sound during summer. Overall, image quality was sufficient to assess molting extent and type for 97.6% (n = 81) of the uniquely identified individuals (n = 83), and revealed that 100% of the individuals had skin irregularities consistent with molting (Fig 8A & S1 Data). We found that molting was extensive for 37.4% of the identified whales, whereby sloughing skin represented >66% of their body. We also found that 37.04% of animals had sloughing skin over 33 to 66% of their body and 25.93% had molting skin on <33% of their body. Over half (58.02%) of the photographed animals had the mottled skin pattern without evidence of rubbing, while 40.74% showed signs of rock-rubbing behavior (Fig 8B).

Our estimated occurrence of rock rubbing from our photographs is likely underestimated due to: 1) limited image quality, particularly when whales were photographed below ~10 m depth; and 2) limited perspective of individual’s body whereby evidence of rock rubbing (i.e., light gray lines) may be on the ventral side of the animal and thus excluded from our analysis. As a consequence, our estimate of the proportion (40.74%) of unique individuals bearing marks from probable rock-rubbing behavior is a minimum estimate.

**Fig 9:**
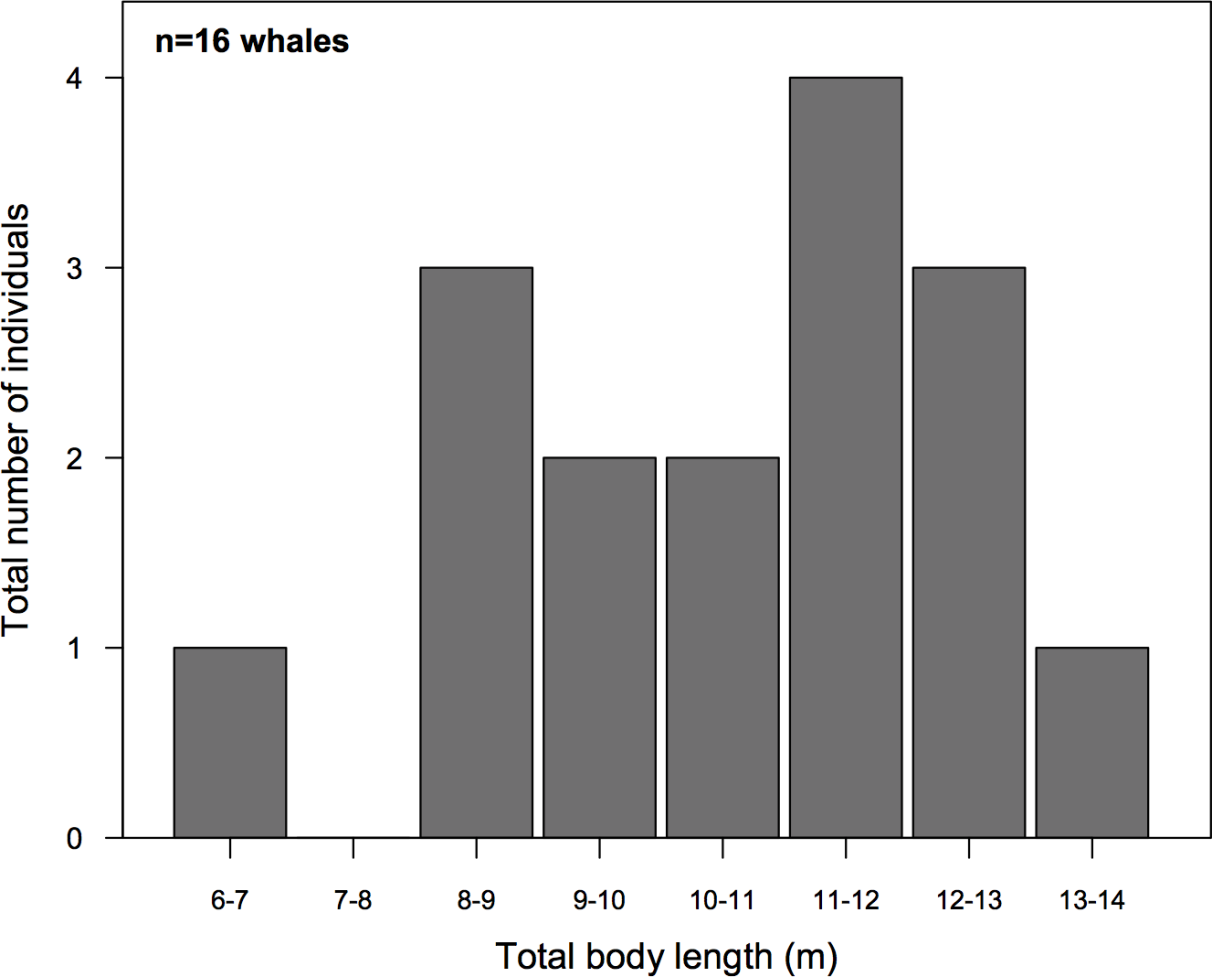
The measured body lengths of 16 unique bowhead whales separated into 8 bins where ~6 m was the minimum size and ~14 m was the maximum. The total body length represents the straight-line distance calculated between the tip of the snout to the fluke notch.

Overall, we measured body lengths for 16 unique whales ranging from 6.3–14.2 m, with a mean length of 10.60 m (± 2.01 SD) (Fig 9). Age-class was broadly inferred based on previous studies [23,28,29] that found calves (i.e., young-of-the year) are ~4–7.5 m in August and September, young juveniles (1 to 8–10 y) are 5.8–10 m [26], sexually immature sub-adults (8–10 to ~25 y) are 10–13 m, and sexually mature adult (25+ y) males exceed 12.5 m [30] and females exceed 13 m [31]. We used the threshold of 13 m for assigning individuals adult status. Of the animals measured, none were calves based on the morphological differences between calves and yearlings [26]. However, we did observe one small yearling measuring 6.26 m. Consequently, we concluded that 38% (n = 6) of the measured animals were young juveniles (8.59 m, ± 1.33 SD), 56% (n = 9) were sub-adults (11.54 m, ± 0.91 SD), and only one animal was an adult (6%) with a body length of 14.22 m. Overall, our measurements of total body length demonstrated that both juvenile and adult animals occupied Cumberland Sound, and that all animals had sloughing skin.

#### Energetic implications of molting

Although we were unable to quantify the proportion of time allocated to molting activities, whales were routinely observed, during daylight hours, resting in addition to actively rubbing against the rocks suggesting that individuals allocate considerable time to these two activities. On numerous occasions, our sUAS deployments documented molting individuals resting in nearshore waters where rock rubbing was previously observed.

Molting is energetically costly for pinniped species that elevate their resting metabolism while increasing blood flow to the epidermis and generating new hair [32–35]. Seals and sea lions have behavioral adaptations to partially offset the metabolic costs associated with molting, such as increasing the time spent on land (i.e., reduced energy expenditure due to thermoregulation and activity costs associated with swimming). Similarly, bowhead whales may adjust their daily activity costs by increasing the proportion of time spent resting. This may be simultaneously beneficial as warmer water may expedite the molting process [4] and taking refuge in shallow, protected bays may mitigate predation from killer whales.

Unlike seals and sea lions that reportedly experience thermoregulatory benefits by hauling out while molting, bowhead whales may overheat while molting in warmer water because they are too well-insulated (bowheads have the thickest blubber of any marine mammal, ranging from 20–35 cm) [36–40] and have small surface area to volume ratios that favor retention of metabolic heat. Maximum daily surface water temperatures ranged from ~4 to 9.5° C in Kingnait Fiord based on the CTD data collected during August 2016, and appeared to be highest near the rock-rubbing habitat with surface temperatures ranging from ~8 to 9.5° C. However, maximum water temperatures encountered by bowhead whales may be even warmer in shallower (~8 m) areas compared with our measurements, which were made in comparatively deep (~100 m) areas of the bay just outside of where rock rubbing occurred. Consequently, bowheads may have to use vascular adaptations to dissipate excess heat while rock rubbing in the warmer, coastal waters.

One way for bowhead whales to dump excess heat may be to use their intraoral thermoregulatory organ located in the root of the tongue (i.e., counter current retial vessels) [41] and at the center of the hard palate stretching to the tip of the rostral palate (i.e., palate rete or corpus cavernosum maxillaris) [36]. They could effectively use this organ by slightly opening their mouths to permit cooling seawater to enter their mouth and flow over the retial vessels in the tongue and the palate rete. We observed several bowhead whales in the aerial images with their mouths slightly agape (S1 Image) near rock rubbing areas (i.e., Brown Harbour). While it is possible that they were feeding, prior prey sampling in similar shallow habitats in Kingnait Fiord found very few zooplankton and their baleen was not visibly extended. It therefore seems more plausible that the whales opened their mouths to cool themselves because they were thermally stressed in the warmer, shallow rock-rubbing habitat while actively swimming during exfoliation activities. They may have thus regulated their body temperature by exchanging heat from enlarged blood vessels in their tongues and palates with the comparatively cooler seawater [36–41].

#### Biological significance of molting

There are biological factors affecting skin condition that may explain why bowhead whales molt. One is that they may slough their skin to shed ectoparasites such as cyamids (i.e., whale lice) [42,43] and accumulated diatoms (i.e., phytoplankton) [44] that may damage their epidermis and potentially impede thermoregulation. Another possibility is that bowhead whales are shedding solar damaged skin [45]. Annual replacement of skin may reduce the risk of extended exposure to ultraviolet radiation during summer in high-latitude habitats [46], and may be particularly important for long-lived species such as bowhead whales because skin damage accumulates with age [47]. Regularly sloughing skin damaged by the accumulation of parasites, diatoms and solar radiation may thus allow bowhead whales to maintain epidermal function and integrity over time.

## Conclusions

Overall, our observation of skin irregularities (e.g., mottled skin pattern, sharp light gray lines, loose epidermis) of various age-classes (juveniles, sub-adults and adults) provides strong evidence that molting is pervasive for bowhead whales during summer in Cumberland Sound. In Cumberland Sound, molting occurred in shallow, warm coastal areas that had low-salinity surface waters (characteristic of sub-Arctic fiords), and appeared to be facilitated by rubbing on large rocks. The elevated water temperature in rock-rubbing habitat may stimulate epidermal growth [11,48], whereby increased water temperature elevates skin temperature and enhances the rate of cutaneous metabolic processes [11]. Furthermore, increased ambient temperatures promotes cutaneous blood flow, bringing nutrients and hormones (e.g., thyroid hormone) known to stimulate epidermal proliferation [11]. Such habitat is comparable to areas where beluga whales rub on rocky substrate in estuaries [24,25], and where bowheads belonging to the Okhotsk Sea population were observed molting [11,15,48].

Our findings lend support to previous hypotheses that molting is facilitated by pronounced changes in oceanographic conditions such as water temperature [18], and suggest that rock-rubbing behavior is used to facilitate the molting process through exfoliation. Additional research needs to address questions regarding the seasonality of the molt (i.e., is molting more pronounced during summer months or does it occur uniformly and continuously throughout the year?) by collecting year-round aerial imagery (excluding winter months with 24 hour darkness), and monitoring the skin condition of bowhead whales over time. Finally, our observations provide evidence that the function of “rock-nosing” observed by whalers, scientists, and northern community members is related to exfoliation to facilitate molting.

## Supporting information

S1 Movie sUAS video of one bowhead whale rubbing on a large rock in Brown Harbour on 7 August 2016. This individual (**A**) was observed with three other individuals that subsequently engaged in rubbing behavior (Fig 6).
(MOV)

S1 Data sUAS data for 83 unique bowhead whales used to determine body length (m), sloughing amount (0 = none, 1 = <33%, 2 = 33–66%, 3 = >66% and <100% and 4 = 100%), sloughing type (0 = none, 1 = light gray lines from probable rock-rubbing behavior, 3 = covered) and whether the mouth was closed (e.g., 0), slightly open (1), wide open (2), or indeterminable (9). (XLSX)

S1 Image Example of a bowhead whale with mouth slightly agape near Brown Harbour during August 2016. (TIFF)
