# Correction: Evidence of molting and the function of "rock-nosing" behavior in bowhead whales in the eastern Canadian Arctic

**DOI:** 10.1371/journal.pone.0192813

**Published:** 2018-02-08

**Authors:** Sarah M. E. Fortune, William R. Koski, Jeff W. Higdon, Andrew W. Trites, Mark F. Baumgartner, Steven H. Ferguson

A reference is omitted from the first sentence of the second paragraph under the subheading “2016 Observations” in the Results and Discussion section and references 22–24 should not have been cited.

The sentence should read: Previous observations of bowhead rock-rubbing behavior have been documented during late summer and early fall in Isabella Bay (Baffin Bay), whereby bowhead whales engaged in “grooming” activities by rubbing on the bottom (Finley, 1990).

The reference is: Finley KJ (1990) Isabella Bay, Baffin Island: An important historical and present-day concentration area for the endangered bowhead whale (*Balaena mysticetus*) of the eastern Canadian Arctic. Arctic 43: 137–152.

A reference is omitted from the last sentence of the second paragraph under the subheading “2016 Observations” in the Results and Discussion section and reference 26 should not have been cited.

The sentence should read: More recently, similar “rock-nose” behavior was observed during an aerial survey of Isabella Bay on 13 September 1979 (Koski, Davis, 1980).

The reference is: Koski WR, Davis RA (1980) Studies of the late summer distribution and fall migration of marine mammals in NW Baffin Bay and E Lancaster Sound, 1979. LGL Limited. Toronto, Ontario. 214 pp.

The last sentence of the first paragraph in the Conclusion section should have cited references 11, 15 and 48 instead of 24 and 25 and reference 18 instead of 11, 15 and 48.

The correct sentence should read: Such habitat is comparable to areas where beluga whales rub on rocky substrate in estuaries [11,15,48], and where bowheads belonging to the Okhotsk Sea population were observed molting [18].
