# Supplementary figures and images for: Evidence of molting and the function of “rock-nosing” behavior in bowhead whales in the eastern Canadian Arctic
Source: PLoS One. 2017 Nov 22;12(11):e0186156. doi: 10.1371/journal.pone.0186156 (PMC5699794; doi:10.1371/journal.pone.0186156)

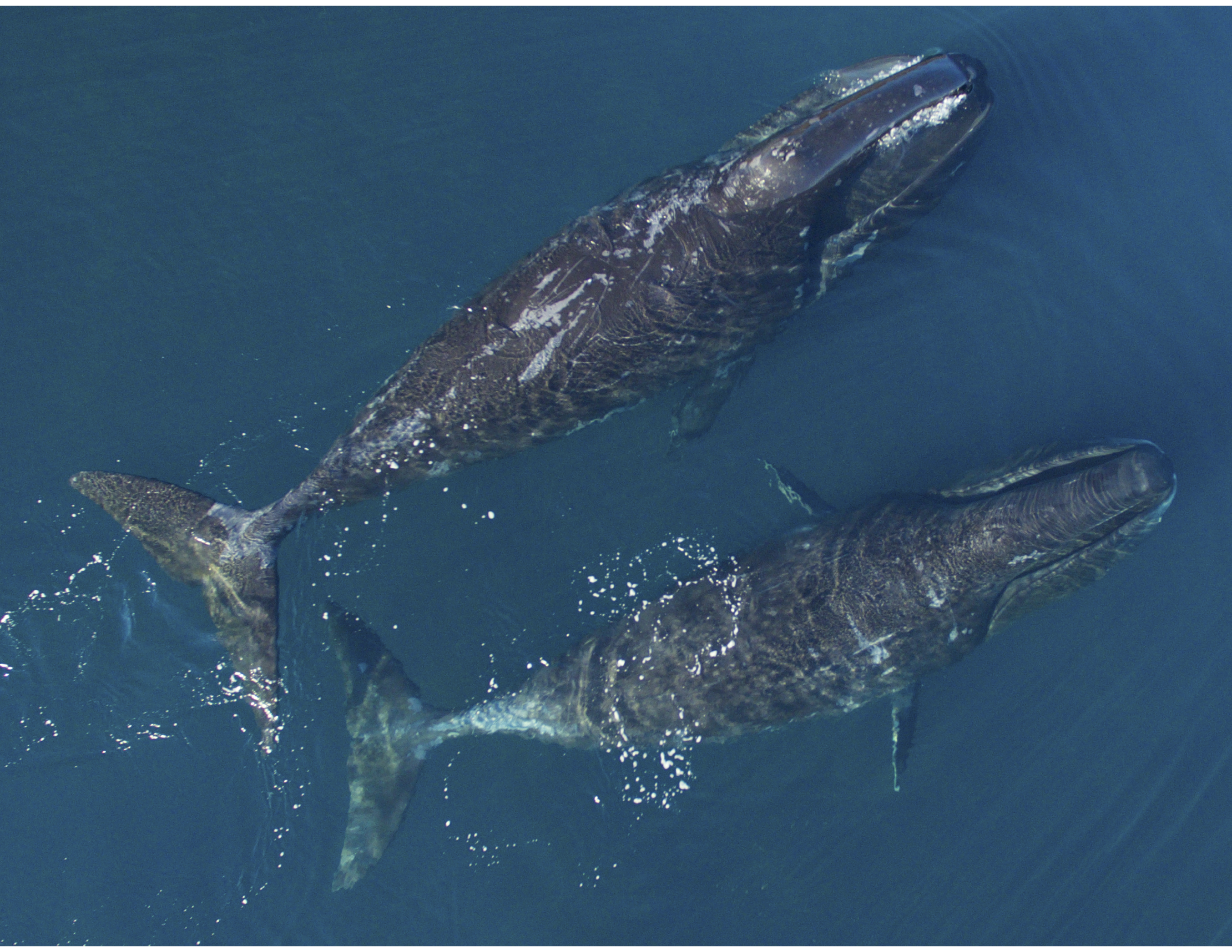

Supplement: S1 Image — (TIFF) [file pone.0186156.s003.tiff]
